# Poxviruses: Slipping and sliding through transcription and translation

**DOI:** 10.1371/journal.ppat.1006634

**Published:** 2017-11-16

**Authors:** Derek Walsh

**Affiliations:** Department of Microbiology-Immunology, Feinberg School of Medicine, Northwestern University, Chicago, Illinois, United States of America; Mount Sinai School of Medicine, UNITED STATES

Poxviruses are truly remarkable pathogens. The most notorious, variola virus (VarV), is thought to have emerged in Africa around 3,000–4,000 years ago. From there, VarV swept across the world causing smallpox, a disease that killed more people than all other infectious agents in recorded history combined [1]. Noticing apparent immunity in milkmaids, Edward Jenner inoculated naïve children with cowpox, resulting in protection against smallpox and ushering in the era of vaccines (“vacca” being Latin for “cow”). Intriguingly, we now use a close relative of VarV, vaccinia virus (VacV), both as a vaccine and laboratory prototype for poxvirus infection. Mysteriously, we don’t know the true origin or natural host of VacV because of the lack of records and way in which vaccines were generated and shared across the globe in the earliest attempts to control smallpox [2]. Despite its mysterious origins, VacV was instrumental in making VarV the only human pathogen to have been successfully eradicated. This medical milestone was possible in part because of the fortunate fact that VarV only infects humans, leaving it with no reservoir in which to hide. Serious threats remain from the potential reintroduction of VarV as well as the ongoing emergence and adaptation of new or zoonotic poxviruses. However, these viruses have also become invaluable tools in oncolytic gene therapy and as vaccine vectors in modern medicine.

As if their clinical history wasn’t striking enough, their mode of replication and level of self-sufficiency is equally remarkable [3]. While other mammalian DNA viruses must reach the nucleus to replicate, poxviruses, together with African swine fever virus (the sole member of the Asfaviridae), replicate entirely in the cytoplasm within compartments called viral factories (VFs). Poxviruses can do this because their large genomes encode hundreds of proteins that include their own dedicated RNA and DNA polymerases, transcription and mRNA biogenesis factors, and their own cytoplasmic redox system. Cleverly, factors controlling early gene expression are generated late in infection and packaged into new virions. As such, upon entry and fusion, poxviruses rapidly transcribe and extrude early mRNAs into the host cell cytoplasm. Synthesis of early viral proteins leads to core uncoating and progression of the viral gene expression program. Remodeling of the endoplasmic reticulum establishes the VF, the site of viral DNA replication and formation of progeny virions. However, despite their incredible self-sufficiency, poxviruses, like all viruses, remain entirely dependent on host ribosomes to translate their mRNAs [4]. This is because, beyond the plethora of regulatory initiation, elongation, and termination factors utilized by their eukaryotic hosts, ribosomes alone consist of approximately 79 protein and 4 rRNA subunits. Thus, encoding their own translation system is something apparently beyond the coding capacity of even the largest viruses identified to date, the mimiviruses, which infect amoeba and fall into the same family of nucleocytoplasmic large DNA viruses (NCLDVs) as poxviruses.

## Translational control in eukaryotes

**Fig 1:**
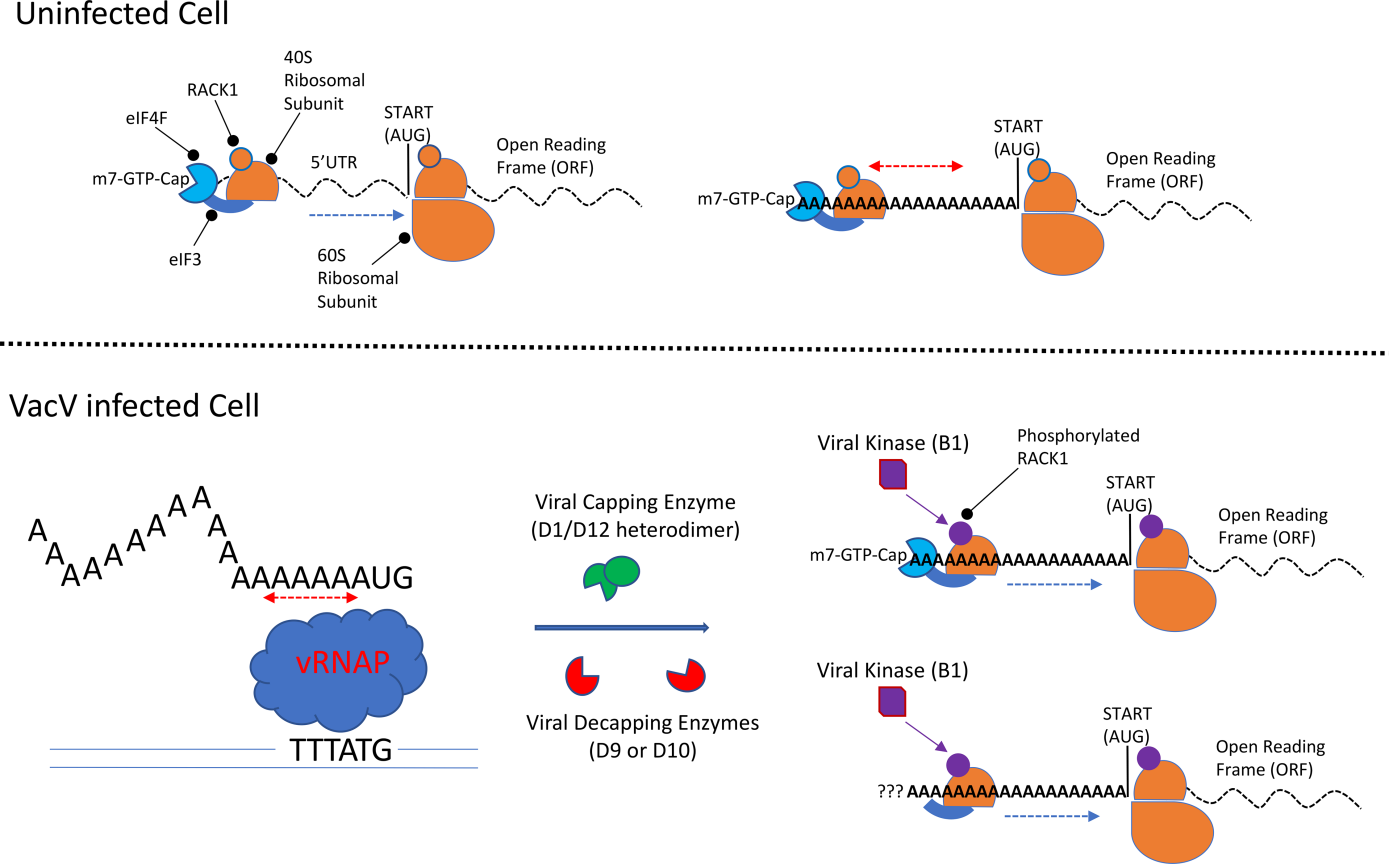
In uninfected cells (top), the 40S ribosomal subunit is recruited to the mRNA m-7-GTP cap through the combined actions of eIF4F and eIF3. The ribosome then scans (blue arrow) the 5′UTR in search of a start codon, at which point the 60S ribosomal subunit joins to initiate translation. Ribosomes slide (red arrows) on polyA stretches, and 5′ polyA leaders do not act as enhancers in mammalian cells. In VacV-infected cells (bottom), the vRNAP slips on intermediate and late promoters, reiterating adenosine residues to create randomly sized polyA leaders. Viral enzymes control mRNA capping and decapping in infected cells, where polyA leaders enable either cap-dependent or cap-independent translation of viral mRNAs; whether cap-independent translation occurs on viral mRNAs that have been decapped or were never capped remains unknown (?). Modification of RACK1 by the viral B1 kinase contributes to the ability of polyA leaders to function in infected cells. eIF, eukaryotic initiation factor; m-7-GTP, 7-Methylguanosine-5’-triphosphate; RACK1, receptor for activated C kinase 1; UTR, untranslated region; VacV, vaccinia virus; vRNAP, viral RNA polymerase.

The vast majority of eukaryotic mRNAs utilize a 7-Methylguanosine-5′-triphosphate (m-7-GTP) cap to mark their 5′ ends and harbor a 3′ polyA tail, both of which influence mRNA stability and translation. To begin translation, ribosomes are recruited by a diverse array of eukaryotic initiation factors (eIFs; Fig 1) [4]. A multiprotein complex called eIF4F binds the 5′ cap and interacts with a second 40S ribosome-associated complex, eIF3. Together, these complexes load 40S subunits on the 5′ end of mRNAs to begin the process of scanning, whereby the 40S ribosome “reads” the mRNA 5′ untranslated region (UTR) in search of a start codon (usually, but not always, an AUG). 5’UTR structure influences scanning and rates of initiation for individual mRNAs. Upon AUG recognition, facilitated by another complex called eIF2, the 60S ribosomal subunit joins to form a translation-competent 80S ribosome. The open reading frame (ORF) is then decoded to produce a polypeptide until such time as the ribosome encounters a stop codon. 3′ UTR sequences, including the polyA tail, can facilitate the reinitiation of translation on the same mRNA. The polyA tail serves a number of other important functions, including stimulating initiation through protein-mediated interactions with eIF4F, as well as functioning in mRNA quality control. In this latter function, if an aberrant mRNA is produced that is out of frame, induces ribosome frameshifts, or lacks a stop codon, ribosome decoding of the polyA tail results in 2 events. First, ribosomes have difficulty with long homopolymeric adenosine stretches and “slide” bidirectionally, reiterating lysines from AAA codons. Secondly, these decoded lysines cause ribosome stalling, signaling decay of the mRNA [5].

## Poxviruses and translational control

The study of poxviruses was fundamental to the discovery of the cap and polyA tail that we now know to be present on most eukaryotic mRNAs. Indeed, poxviruses encode their own capping, decapping, and polyadenylation enzymes [3]. While capable of producing their own mRNAs, poxviruses go to incredible lengths to gain control of host ribosomes needed for their translation. In self-defense, host cells go to equivalent lengths to prevent this. A major host antiviral response involves the inactivation of eIF2 by protein kinase RNA-activated (PKR), leading to widespread suppression of translation [4]. To evade this, poxviruses encode several proteins that either directly target PKR, limit the production of double-stranded RNA (dsRNA), or shield dsRNA from detection [4, 6, 7]. In an incredible form of evolutionary arms race, PKR can adapt to new virus-encoded antagonists. In response, the VacV genome can amplify multiple copies of the K3L gene, allowing for random mutations as a means to explore counteradaptation [8]. Once an adaptive fitness that potently counters PKR emerges in a K3L gene copy, the “gene accordion” can collapse again to retain the newly adapted PKR antagonist. Poxviruses have also been found to activate host signal pathways that stimulate eIFs, enhancing viral protein synthesis and countering host interferon production [9–11]. Although poxviruses suppress host translation through mRNA decapping and other strategies, a limited number of host mRNAs are selectively retained on ribosomes to maintain the synthesis of proteins that perform important tasks, such as cellular energy production [12]. As such, poxviruses are clearly master manipulators of their hosts’ translation system.

While poxvirus mRNAs broadly resemble those of their host, one unusual feature of postreplicative or late-stage mRNAs is the presence of 5′ polyA leaders, or “polyA heads” [3]. Synthesis of postreplicative mRNAs requires an intact TTT sequence motif at the transcription start site, and substitution of even a single T residue drastically impairs transcription [13]. However, the viral RNA polymerase slips at this TTT motif, resulting in reiteration of adenosine residues, thereby forming the polyA leaders that immediately precede the translation start codon in poxvirus postreplicative mRNAs (Fig 1). While randomly generated and at times exceeding 50 nucleotides in length, these leaders average 12–30 nucleotides, studies suggest [3, 14, 15]. For decades, why poxviruses do this and whether these leaders have any biological function has remained enigmatic. Earlier studies suggested they enable translation initiation in the absence of eIFs in vitro [16], although poxvirus protein synthesis in infected cells exhibits varying degrees of sensitivity to eIF4F perturbation [17–19]. While high abundance of viral mRNAs might contribute to apparent eIF4F independence, recent findings show that polyA leaders do indeed confer the ability to employ either cap-dependent or cap-independent modes of initiation but, importantly, do not function as internal ribosome entry sites (IRESs) commonly used by RNA viruses [4, 20]. This capacity for dual modes of initiation likely maximizes the competitiveness of viral mRNAs for ribosomes or may allow them to initiate despite the presence of 2 virus-encoded, indiscriminate decapping enzymes (Fig 1). After ribosome loading, however, these leaders would appear to be an unwise choice because adenosine runs of 11 nucleotides or more cause bidirectional sliding of ribosomes, also dubbed “phase-less wandering” [5, 16]. Intriguingly, mRNAs with polyA leaders show no translational advantage in uninfected cells, yet their translation is enhanced in VacV-infected cells, suggesting that infection modifies the host environment to accommodate viral mRNAs [20, 21]. In a remarkable form of viral “customization” of ribosomes, it has recently been revealed that the VacV kinase B1 phosphorylates residues in a flexible loop in the small ribosomal subunit protein, receptor for activated C kinase 1 (RACK1) [21]. This appears to slow initiation rates to facilitate leader activity, potentially compensating for sliding and/or allowing cap-independent viral transcripts to dominate. This finding also begins to illuminate yet another long-standing mystery: polyA leaders and the tobacco mosaic virus (TMV) “Omega Leader,” which consists of CAA repeats, show little or no activity in mammals but act as translational enhancers in plant cell extracts [20–23]. Why this is, and why poxviruses that replicate in mammals would produce such leaders, has remained unclear. However, although RACK1 is structurally highly conserved, the loop region targeted by VacV varies between species. Notably, in plants, the loop contains naturally negatively charged amino acids that are not present in mammals. Through unique phosphorylation events in human RACK1, poxviruses mimic the charged state found in plant RACK1 [21]. While some of their mysteries have begun to be solved, there is undoubtedly much more to learn about how these enigmatic elements function. Moreover, what might once have been seen as the random generation of odd leaders through “erroneous” slippage of the viral RNA polymerase is now clearly part of a well-orchestrated strategy to confer translational advantages to viral mRNAs. Curiously, both transcriptional and translational process hinge on nucleotide sequences that cause viral RNA polymerase or ribosome slippage, but the mystery of how this unusual coupled strategy evolved may prove the most challenging of all to illuminate.
