# Correction: Poxviruses: Slipping and sliding through transcription and translation

**DOI:** 10.1371/journal.ppat.1006832

**Published:** 2018-01-04

**Authors:** Derek Walsh

There is an error in reference 2. The correct reference is: Damaso CR. Revisiting Jenner’s mysteries, the role of the Beaugency lymph in the evolutionary path of ancient smallpox vaccines. Lancet Infectious Diseases. 2017;S1473-3099(17)30445-0.
